# Tool use as distributed cognition: how tools help, hinder and define manual skill

**DOI:** 10.3389/fpsyg.2014.00116

**Published:** 2014-02-24

**Authors:** Chris Baber, Manish Parekh, Tulin G. Cengiz

**Affiliations:** ^1^School of Electronic, Electrical and Computer Engineering, University of BirminghamBirmingham, UK; ^2^Department of Industrial Engineering, Uludag UniversityBursa, Turkey

**Keywords:** distributed cognition, tool use, affordances, representation, extended mind, systems dynamics

## Abstract

Our thesis in this paper is that, in order to appreciate the interplay between cognitive (goal-directed) and physical performance in tool use, it is necessary to determine the role that representations play in the use of tools. We argue that rather being solely a matter of internal (mental) representation, tool use makes use of the external representations that define the human–environment–tool–object system. This requires the notion of Distributed Cognition to encompass not simply the manner in which artifacts represent concepts but also how they represent praxis. Our argument is that this can be extended to include how artifacts-in-context afford use and how this response to affordances constitutes a particular form of skilled performance. By artifacts-in-context, we do not mean solely the affordances offered by the physical dimensions of a tool but also the interaction between the tool and the object that it is being used on. From this, “affordance” does not simply relate to the physical appearance of the tool but anticipates subsequent actions by the user directed towards the goal of changing the state of the object and this is best understood in terms of the “complimentarity” in the system. This assertion raises two challenges which are explored in this paper. The first is to distinguish “affordance” from the adaptation that one might expect to see in descriptions of motor control; when we speak of “affordance” as a form of anticipation, don’t we just mean the ability to adjust movements in response to physical demands? The second is to distinguish “affordance” from a schema of the tool; when we talk about anticipation, don’t we just mean the ability to call on a schema representing a “recipe” for using *that* tool for *that* task? This question of representation, specifically what knowledge needs to be represented in tool use, is central to this paper.

## INTRODUCTION

The central question for this paper is what representations are employed when using tools? In this paper, the term “representation” is taken to mean a set of parameters which describe an action (from goal to execution). In broad terms, one answer to this question might see the set of parameters as being specified prior to an action being performed, e.g., in the form of an action schema, or as being recruited in preparation of the action, e.g., in the form of activation of specific brain regions. In this case, the question becomes one of identifying what the representation might contain and where it might be stored. This is what we refer to as an “internal representation.” Alternatively, the parameters might arise from the performance of the action in response to constraints imposed by the environment, e.g., in the dynamic behavior of a system. This is what we refer to as an “external representation.” We argue that, while there is evidence to support the view that tool use can be guided by “internal representation,” this only provides a partial view of such activity and that the use of “external representation” can provide a viable alternative account.

The position taken in this paper assumes that the physical behavior of the person can be viewed as part and parcel of their cognitive activity, and that there is a close coupling between a person’s action and their perception of features of objects in the world. However, neither assumption fully captures human activity when using physical objects for goal-directed activity (which is the broad definition of tool-use employed in this paper). Thus, we argue for a broader appreciation of Gibson’s (1979) notion of *complimentarity* as an explanation of affordance at a “system” level. The notion of “system” here draws on Maravita and Iriki’s (2004) idea of the “hand-tool body schema” but we extend this to cover person–environment–tool–object. For us, this requires the notion of Distributed Cognition to encompass not simply the manner in which artifacts represent concepts but also how they represent praxis. In other words, the design of the tool (as a human-made artifact) reflects not only the manufacturing process but also a set of assumptions about how that tool should be grasped and manipulated, and how activities involving that tool can be performed “correctly.” This means that “tools” are distinct from other physical objects in the human environment because their use is defined not only by their appearance or the user’s goals but also by cultural constraints that have influenced their production (Baber, 2003, 2006; Burghardt et al., 2011). While there are instances in which other physical objects, such as sticks or stones, can fulfil tool functions, and while the neurological evidence suggests that images of these objects activate similar regions in the brain to images of tools, there is accumulating evidence that the pattern of brain activation for tools is somewhat different from that of physical objects *per se.*

*“Human beings, viewed as behaving systems, are quite simple. The apparent complexity of our behaviour over time is largely a reflection of the environment in which we find ourselves”* [Simon, 1969]

While Simon was not talking explicitly about Distributed Cognition, this quotation points to the need to understand human behavior in the environment in which it occurs. For us this implies a need to better understand how the environment makes an impact on our actions and decisions, and this suggests the benefit of an approach which studies human action as they occur in natural (or as near natural as possible) conditions. This raises challenges for “ecological validity” (Neisser, 1967) which takes us out of the laboratory (or, for that matter, the brain scanner) and into the settings in which activity is performed. A primary reason for this quest is the assumption that the relations between human, environment, tool, and object are fundamental to the study of perception and action (Gibson, 1979; Beek and Bingham, 1991; Newell, 1991). A study of the activities of tool use away from typical environments runs the risk of ignoring the constraints that the environment places on the performance of these activities. Thus, it is vital to ensure that enough of the characteristics of the person–environment–tool–object system are reflected in the design of studies (even if these are conducted in laboratories). We are interested in ways in which we might be able to capture data from the tool using actions of people in work environments, through analyzing video of their activity (and discussing these videos with them) or through putting sensors on the tools that they use. For this paper, the focus will be on the use of data collected from sensors on tools. Two areas of activity will be used in this paper: using hand-tools in jewelery and eating with cutlery. In both areas, the concern will be to compare experienced and less experienced users of the tools. The comparison will be qualitative rather than quantitative, i.e., examples of the data collected during our studies will be presented but more detailed analyses of these data will be found in other papers.

### WHAT NEEDS TO BE REPRESENTED IN TOOL USE?

By way of a definition of the word “tool,” we propose that a tool is a physical object which lends itself to manipulation by a human (or animal) in order to solve a problem presented by objects in the physical environment. This notion of tool-use as a form of problem-solving not only emphasizes the goal-directed aspect of using tools but also the need to respond to, and overcome, constraints. This definition allows us to combine both the physical action of manipulating the tool with the cognitive aspects of goal-directed, purposeful behavior. Following a similar line of argument (tool use as problem solving), Osiurak et al. (2010) suggest that the coordination of the physical actions involved in using tools represent a problem to be solved. They view cognition and physical activity in a dialectic in which a particular goal encourages the perception of particular affordances in the world and serves to influence the bodily action to perform, which, in turn, moves the person towards their goal. This strikes us as an elegant reformulation of the notion of affordance as a goal-directed, physical response to the environment. The difference between this view and the one presented in this paper is simply (we believe) a matter of scale: rather than considering problem solving in the broad terms that Osiurak et al. (2010) offer, our focus is on the interface between tool and object (or, rather, we propose that the “problem” that concerns tool users is how to modify the object in ways that satisfies a goal, given the constraints that the tool (and the tool-users’ ability to wield that tool) might impose on their action).

In order to explore further the question of representation in tool use, it is important to consider *what *needs to be represented in order to use a tool. Tool use is not only a matter of recognizing that an object is a tool but also of knowing how to hold and manipulate that particular tool. It is also a matter of understanding the consequences of a particular way of using a particular tool. Knowing that a piercing saw (used by jewelers to cut metals) is held vertically for cutting (with the wrist more or less locked and most of the motion about the elbow), and has teeth which cut in one direction, leads to an understanding that the cut is made on the downstroke (not the upstroke), and helps define a set of possible actions when using this tool. From this it might appear that we are arguing for (at least) some representations of the tool and the actions associated to be internal to the person. Does this mean that these representations are stored in the brain?

## INTERNAL REPRESENTATION: NEURAL ACTIVATION IN TOOL USE

The suggestion that the use of tools depends on “internal models” is nicely encapsulated in a recent paper by Imamizu and Kawato (2012). They review literature and report studies which indicate the existence of both a feed-forward model, taking efference copies of motor commands to enable motion dynamics, and inverse models used to manage these dynamics. During learning, changes in cerebellar activity indicate the acquisition and refinement of such models. As we argue in this paper, the notion that brain-based “internal models” are *causal* represents a particular view of tool use, and we are proposing that it is possible to explain much of the activity involved in tool use through a combination of Distributed Cognition and dynamics which might not be represented in the brain *per se*. However, before exploring this proposal further, we consider some of the neuropsychological evidence relating to tool use. Imamizu and Kawato (2012) review neuropsychological studies of tool use and suggest that, *“[A]lthough the brain regions related to each type of component cannot be uniquely determined…”* (p. 325) there are two distinct functional regions of the brain related to tool use: one related to the physical skills involved in dextrous tool manipulation, and one related to the semantic and conceptual knowledge relating to the functions of tools (see also Lewis, 2006; Higuchi et al., 2007, 2009). These distinct regions are discussed in more detail in the rest of this section.

In their now classic study, Chao and Martin (2000) used functional magnetic resonance imaging (fMRI) to show that viewing and naming of tools led to activation of the left ventral premotor cortex, suggesting a strong relationship between the physical appearance of objects and the fact these objects could be acted upon. Grafton et al. (1997) used positron emission tomography (PET) scanning of participants asked to observe or (silently) name tools and their use. Observation of tools resulted in strong activation of the left dorsal premotor cortex, and (silent) naming of these tools resulted in additional activation of Broca’s area. However, naming the *use* of the tools led to activation in Broca’s area, together with activation in left dorsal premotor cortex, left ventral premotor cortex, and left supplementary motor area. This implies that naming the use of a tool (even when the action is not performed with it) has motor valence which is additional to that obtained when looking at the tool. It also suggests that the physical appearance and name of a tool activates slightly different areas than the use of the tool. Taken together these, and related, studies imply that brain activation relates to specific properties of the tool-as-form and tool-as-function, and that these properties are not solely related to a tool’s physical appearance but also to how it moves or how it is used (Johnson-Frey, 2004; Martin, 2007).

One suggestion is that representations of tools are held in specific regions of the brain and become activated during activities in which similar objects are used. According to Gallivan et al. (2013) the distributed coding of different actions associated with hand movement and tool use imply that these actions are represented separately and then integrated in the frontoparietal cortex. As Yee et al. (2013) show, in an ingenious experiment, asking people to think about manipulable objects when they are performing manual actions which are incompatible with those objects is difficult (but it easy to think about non-manipulable objects during the performance of such actions). This suggests that the meaning of objects (specifically in terms of their properties which support manipulation) is recruited during action, and that incompatible action interferes with this. Furthermore, work by Hoeren et al. (2013) points to the suggestion that the recognition of action (performed by other people) is processed using distinct streams: the dorso-dorsal stream focusing on movement determined by the properties of the objects being used, and the dorso-ventral stream focusing on functional appropriateness and dexterity of task performance.

### KNOWLEDGE OF (FAMILIAR) TOOL USE

The discussion so far points to the need to draw on knowledge of the appropriateness of a given tool for a given task and how to wield that tool to achieve the most effective result. Riddoch et al. (2006) presented patients (manifesting visual extinction) with images of pairs of objects. The pairs showed objects which people are likely to have experienced being used together (e.g., a bottle and a glass), or objects which could plausibly be used together, although might not have been experienced as such (e.g., a bottle and a bucket), or were randomly paired in order to, as far as possible, produce pairs which had no association. The results showed that commonly paired objects were identified more quickly than plausibly paired objects which, in turn, were identified more quickly than the randomly paired objects (although this latter finding only held when the image showed the objects being used together rather than having them presented side by side). One implication of this work (which could be applied to normals as well as patients) is that the common and plausible pairs activate familiar routines in tool use. In contrast with this observation, Vingerhoets (2008) found that presentation of images of “familiar” or “unfamiliar” tools activated the same brain regions, with “unfamiliar” tools generating more activation in the left hemispheric medial posterior occipital and inferior posterior temporal areas (in comparison to images of “familiar” tools) and more activation around the supramarginal gyrus for the familiar tools. While these results showed strong individual differences, they also imply that the activation in response to “familiar” tools can be associated with knowledge of the appropriate hand position for the *use* of the tool (as opposed to simply whether or not the tool *could* be grasped).

A similar line of argument comes from studies in which participants are asked to pick up handled objects (such as cups) when the handle faces either towards or away from the hand that they are instructed to use (Tucker and Ellis, 2001). For example, Bub et al. (2012) presented images of everyday objects together with images of hands in different orientations. The objects all had handles which were either oriented horizontally, e.g., pliers, frying pan, or vertically, e.g., beer mug, hairdryer. Participants were asked to name the object. Reaction (naming) time was significantly faster when both hand and wrist orientation matched the type of handle, or when neither hand and wrist orientation matched the handle, but much slower when either hand or wrist orientation was incongruent. Relating this to the previous discussion of neural imaging, one can assume that the photographs of the hands and the objects might have activated different regions, with a combination occurring *prior* to response.

The suggestion that there might be preparative neural activity which corresponds to different types of action (Rizzolatti et al., 1988) could provide evidence for the recruitment of a set of representations determining task performance. Certainly the movement-related cortical potential (MRCP) recorded from electroencephalography (EEG) begins 2–3 s before the onset of movement (Toma and Hallett, 2003; Wheaton et al., 2005). Furthermore, onset seems to be proportional to complexity of movement, with more complex movements having longer onset times. Such activity, typically in the left posterior parietal cortex, is taken to indicate the need to manage complex motor activity and, as Wheaton et al. (2005) propose may include *“…imagining executing such movements; the goal of the movement; determining the natural position and setting required for proper performance; sequence of motor acts and comprehension of the task.”* (p. 535). While we have every reason to accept that complex movements involve recruitment of appropriate muscle groupings and specification of appropriate control parameters, we do not see why this necessarily involves the definition of specific representations of the task context. Thus, our debate is not with the neurological evidence *per se *but with the assumptions that these *must *point to internal representations which drive behavior.

What is interesting in the Bub et al. (2012) study is less the reinforcing of activation of congruent images (or, indeed, the effect of incongruence) than the problems caused when one of the hand images did not match the other image or the object. Bub et al. (2012) suggest that this reflected disruption of the plan being developed in working memory (with the images activating particular judgments about using tools). However, the images presented in these studies serve as the (external) representations about which people are asked to make judgments. As such the idea that they would need to create corresponding internal representations in order to make such judgments seems a little odd. The images that are presented provided sufficient information to make a judgment and the need is to determine whether these are “true” or “possible.” On the one hand, it seems plausible to assume that prior experience provides the “grounding” (Mizelle and Wheaton, 2010) of a tool in terms of its usage, but on the other hand, it is equally plausible that this could be part of the person’s action repertoire (e.g., in terms of Bernstein’s (1967) idea of coordinative structures) as it is activation of specific regions of the brain.

For an action in which participants had to use different tools to touch a target, precuing the target had no benefit on performance, but precuing the tool to use had significant benefits (Massen and Prinz, 2007). We take this to suggest that the precuing of the tool enabled the recruitment of the appropriate “coordinative structure,” to use Bernstein’s (1967) phrase describing combinations of muscle enervation and limb movement, to perform the task with a given tool. What is interesting about this interpretation of their findings is that “representation” need not be same for different tasks (and, we would argue, shows how it can shift to outside the brain *per se*). Hermsdörfer et al. (2006) compared performance of apraxic patients with a control group of normals on a sawing task. Participants were asked to demonstrate sawing under three conditions: when they were shown a photograph of a saw and asked to pantomime sawing; when they were shown the photograph, given a piece of wood (the same size as the saw’s handle) to hold and asked to pantomime sawing; when they were given the actual saw to hold. While the controls showed fairly consistent performance across the three conditions, apraxic patients showed motion errors (deduced from 3D motion tracking) in the first two conditions. Typically, these errors involved substituting mediolateral motion for the anteposterial motion expected. Interestingly, these errors were *not *apparent when the apraxic patients were given the actual saw to use. On the one hand, this supports a common finding in apraxic studies (that providing people with the physical object seems to enable them to perform tasks more effectively and reliably than when they do not have the object to hand). On the other hand, we believe it tells us something about the need for internal representation when using tools. Hermsdörfer et al. (2006) conclude that *“… pantomiming the use of a tool and actually using the tool are facilitated by largely different neural processes which differ in demands and goals.”* [p. 1651]. We would argue further that these differences arise because the use of the tool involves the control of the person–environment–tool–object system and need *not* depend on internal representation.

### CONCLUSION

Just because the tool-using behaviors have neural correlates does not mean that these are the only places in which representations for the behaviors exist. Clearly, the type of grasp is likely to be influenced by the action which one intends to perform with the tool. We have a repertoire of appropriate grasps for manipulable objects, and we adapt these grasps according to contextual demands. The adaptation often occurs with sufficient fluency and speed to make it unlikely that we have simply retrieved a particular piece of “motor schema” from memory and applied this; indeed, the very notion of a “motor schema” (with its attendant implication of stored sequences of action) has been called into question (Sherwood and Lee, 2003; Shea and Wulf, 2005)*. *Thus, we argue the tool user is, partly, using the tool to make changes to objects in the environment, but also partly using the tool to help create further opportunities in the environment for using the tool. In other words, tool use is an interplay between seeking a defined goal and managing the affordances arising from changes in the object in the environment (resulting from the ongoing use of tools). Before discussing the collection of data and their analysis, the next section describes the particular stance taken in this paper: Distributed Cognition.

## EXTERNAL REPRESENTATION: DISTRIBUTED COGNITION AND THE EXTENDED MIND

As the phrase implies, Distributed Cognition addresses situations in which the processing of information occurs outside the brain. For some writers, this is the proposal that the environment and the objects it contains can shape the way in which cognition is performed (Zhang and Norman, 1994; Hutchins, 1995a,b; Scaife and Rogers, 1996). While this position could be seen as paraphrasing the well-known observation that the representation of a problem space influences the strategy that problem solvers apply (Chase and Simon, 1973; Larkin et al., 1980; Chi et al., 1981), e.g., changing the layout of a puzzle can make it easier or harder to solve, it also points to the importance of interactivity in behavior. For example, people playing Tetris or Scrabble can benefit (in some situations) by being allowed to manipulate and rearrange the playing pieces (Kirsh and Maglio, 1994; Maglio et al., 1999). This points to the need to not simply focus on the arrangement and design of the problem representation, but also on the nature of the interaction between person and objects. From this point of view, “embodiment” becomes an essential feature of acting not only on the objects but also on the cognitive tasks involved in problem solving. In other words, rearranging the pieces is not simply performed in order to assist thinking, it *is *thinking. This is taken to mean that the relationships within the human–environment–tool–object system not only supports (or affords) different actions but also shapes cognition (Wilson, 2002). The reason for this is that activity within this system is often time-limited, in that the actions are performed at speed, in real-time and offer little opportunity for planning (what Clark, 1997, has termed “mind on the hoof”). From this, the main purpose of cognition (in tool use) is to support action in as situation-appropriate manner as possible. It also suggests that, rather than needing to construct “internal representations” of the environment, it is sufficient to respond to the appearance of the environment. From his work with robots, Brooks (1990) pointed out that robot performance could be more efficient if they spent less time “planning” and creating representations, and more time “acting” because *“the world is its own best model”* [Brooks, 1990, 12].

### CONSIDERING AFFORDANCE

Our reading of Gibson’s (1977, 1979) concept of affordance lies in his notion of “complimentarity” in which the properties of objects in the environment are responded to by the animal. Turvey (1992) offers the term “effectivity” as a way of capturing these properties of the animal. So, one way of seeing affordance lies in the complimentarity between the object’s properties and the animal’s effectivity. One of the problems that the idea of “effectivity” and “properties” raises is the suggestion that these are separate aspects which are brought together during the performance of a task, which implies that they are independent, autonomous features which become coupled during task performance. Indeed, Gibson (1979) suggests that the “affordance” exists whether or not the observer perceives or attends to it. If this is the case, then it makes sense to assume that one aspect of the “effectivity” of the task performer would be the neural representations of the actions involved in performing the task (as well as morphological features and motor skills).

There are many situations in which the observer cannot but attend to the affordance, e.g., perseveration in the behavior of stroke patients, or response to “fake” cues by animals. Rather than implying (as Gibson seems to) that the “affordance” is an invariant property of the environment, the fact that perseveration is an unusual state of affairs suggests that humans (and some animals) are able to *choose* to respond to affordances (and by implication, to *see* affordances in different situations). This implies that what is essential to “affordance” is this combination of the property of the object in the environment and the effectivity of the specific individual (with specific knowledge, skills, abilities, and goals) in that environment. While the properties of the object in the environment may well be invariant, the actual**affordance arises from the complimentarity of environment and actor. Affordance is partly a matter of perception-action coupling and partly a matter of intention (goal) – action coupling. Perhaps a better way of putting this is that perception-action coupling is mediated by the intentionality of the actor. However, as Chemero (2009) points out, the idea of separable components that can be coupled runs counter to the notion of complimentarity; taking affordance as the result of the system created by person–environment–tool–object (as we do in this paper) leads to the conclusion that this is a system which is non-decomposable and which exists only during the performance of the given task. From this, we suggest that the goal, in the person–environment–tool–object system is *partly* held by the person (in terms of the effect that they intend to produce on the object) and partly situated (Suchman, 1987) in the ongoing interactivity in the system. This assumption echoes the earlier assertion of van Leeuwen et al. (1994) that tool use can be *“… defined as performing an action on a target by performing an action on a tool. The action on the tool is embedded in the action on the target.”* [van Leeuwen et al. (1994), p. 188–189]. For van Leeuwen et al. (1994) this embedding reflected a “higher-order affordance structure” of “mutually constraining complimentarities.”

van Leeuwen et al. (1994) argued that it was important to understand the role of context in task performance in terms of a *sufficiency principle*, i.e., *“if an affordance has already been realized, there is no need to take it into account.”* [van Leeuwen et al. (1994), p. 190]. To take this a little further, Turvey (1992) suggests that affordance might play a role in “predictive control” of activity and, while the analysis (and indeed use of the term), in this paper might differ from his, the idea that affordance refers not only to immediate action but to future actions is central to the ideas in this paper. Additionally, Mizelle et al. (2013) discuss the notion of *functional *affordance, in which there is an optimal manner in which a given object can be used to achieve a desired goal. For example, Mizelle et al. (2013) note that a hammer can be held a variety of grasps (some involving the handle, some involving the head, for instance) but that there is a grasp which *“… best affords the action of driving a nail…”* (p. 280). This can be seen as taking the predictive control further, in that there is a goal state against action can be optimized. While these notions of affordance *could* be represented internally (in terms of specific neural correlates of functional affordance that can be adapted to contextual demands), the notion of complimentarity followed in this paper offers a more parsimonious explanation. In other words, the Gibsonian notion of affordance is taken in this paper to describe a particular form of complimentarity in the person–environment–tool–object system, and it is the “system” as a whole which can be said to optimize the tool-using activity.

### TASKONOMY AND HOW THE ENVIRONMENT AFFORDS SKILLED ACTION

**FIGURE 1:**
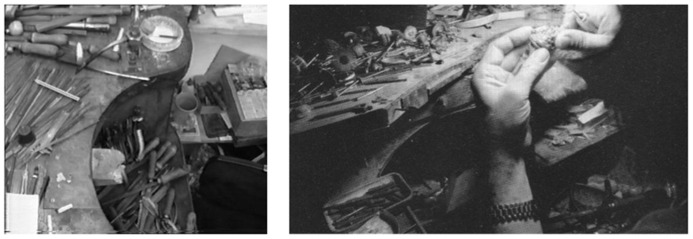
**“Taskonomy” in jewelers’ workspaces**.

One way in which the environment can be created to provide affordances for future action is in the ways in which experts lay out their workspace. In their discussion of blacksmiths, Keller and Keller (1996) use the term “taskonomy” to refer to the ways in which an expert’s knowledge of the tasks to be performed help create the arrangement (taxonomy) of tools in their space. This arrangement is not simply a matter of having particular types of tools kept near each other, but arises through a combination of tools and actions. A similar pattern can be seen in the workspace of the jeweler (**Figure 1**).

As the jeweler performs a particular task, so a tool is picked up, used and then laid down in the workspace; as work progresses so tools are either reused or new ones introduced. However, the expert is often able to describe what work had been completed in a particular workspace by looking at the collection of tools in the immediate vicinity. In some cases, specialized tools will be brought to the workspace with the intention of supporting a particular goal. Thus, the workspace becomes managed to provide particular affordances (in terms of available tools and the position in which these tools are placed to support particular types of grasp). This suggests the anticipation of tasks and the arrangement of the workspace in line with these anticipations. In these ways, the movement of tools in the workspace (as the result of deliberately selecting these in preparation of a specific job, or as the result of picking up and putting down the tools during the performance of the job, or as the result of moving tools which are no longer needed further away from the central point of reaching) becomes part of the structuring of the workspace. Rather than simply reflecting the ebb and flow of actions in the workspace, we argue that this reflects the management of potential affordances and, as such, is a form of Distributed Cognition. The suggestion that moving tools around the workspace is a form of “cognition” is logically the same as the suggestion that presentation of a problem will “frame” the approach to the solution and that manipulating pieces in a puzzle might be a form of thinking. In other words, layout of the workspace will frame the actions which are most likely to be performed and this framing is the result of deliberate choices made to retain, discard or move a tool after it has been used (rather than merely a consequence of moving tools around).

## WORKING WITH TOOLS

In his discussion of craftwork, David Pye draws the useful distinction between “certainty” and “risk” in craftwork. He argues that in “the workmanship of certainty” there is an impetus to design work to ensure consistency, repeatability, and minimize variation or ambiguity. Such work involves heavily proscribed procedures and measures of quality and could be interpreted in terms of industrialized production processes. In this approach, the artifact being produced will be tightly specified prior to production and the resulting artifact will be considered in terms of this specification. Anyone who has constructed flat-pack or self-assembly furniture will have encountered a situation in which the manufacturer has sought to encourage workmanship of certainty. However, anyone who has built self-assembly furniture will also recognize the challenges that this poses. Misreading the instructions or believing that you know what you are doing so don’t need to read the instructions *can *lead to results which differ from the goal. This could be quite minor (a handful of left over components) or quite major (the door which doesn’t open, the shelf which drops out when the unit is stood up). This variation illustrates the workmanship of risk. This, in turn, reflects the variability in outcome which can arise from decisions made by the worker during the performance of the tasks. The decisions could reflect a choice of tool, or knowledge/skill in the use of the tool, but they could equally reflect responses to the opportunities presented (or constraints created) by the materials being used. For example, the knot in a piece of wood, or the finish on one side of the self-assembly wardrobe, could constraint the actions which are possible or could suggest an appropriate action to perform. In contrast, the “workmanship of risk” does not involve such tight specification, i.e., *“… the quality of the result is not predetermined, but depends on the judgment, dexterity and care which the maker exercises as he works.”* (Pye, 1968, p. 20). Rather than the intent or purpose being predetermined, it is now something which crystalizes through the developing interaction between craftworker, tools, and materials being worked. This is something which we noted in our study of jewellery making (Baber and Saini, 1995): the jeweler worked to very sketchy “plans” but adapted these plans to suit the resulting state of the material, often modifying a particular ring or brooch to capitalize on a particular facet that they noticed as the metal was being worked.

*“First, the experienced worker usually employs “smoother*” *and more consistent movements…Secondly, the experienced worker operates more rhythmically, indicating that a higher degree of temporal organization has been achieved. Thirdly, the experienced worker makes better use of the sensory data…Fourthly, the experienced worker reacts in an integrated way to groups of sensory signals, and makes organized grouped responses to them”* [Seymour, 1972, 35–36]

The quotation from Seymour (1972) indicates how the output of the human–environment–tool–object system is being optimized, but not necessarily how the dynamics of the system relate changes in input to output. In order to consider this, we turn our attention to series of studies conducted by Bril and her colleagues, focusing on tasks involving hammering (either stone hammers to knap flint or metal hammers to shape stone or glass beads).

### SYSTEM DYNAMICS: TRANSFORMATIONS IN TOOL USE

Our actions, when using tools, involve the coordination of a set of transformations (Biryukova and Bril, 2012). We transform kinetic energy into tool motion – but need to appreciate how much energy to exert in order to produce the desired motion of the tool (and in order to produce the desired effect on the object from the tool’s motion). We manage dynamic transformations, balancing the movement of the tool in the air and on the object with our own motions and with the outcome of the tool’s activity. We anticipate what effect the tool’s motions will produce and relate these to the outcomes that we desire. As Ingold (2000) points out, *“Intentionality and functionality are … immanent in the activity itself, in the gestural synergy of human being, tool and environment.”* (Ingold, 2000, p. 352.) The ability to both anticipate the outcome of the tool’s action and manage the functionality of the tool are an integral part of the use of the tool. The dynamics of using the tool thus becomes far more important than might be implied by the neurological imaging work which concentrates on the form and function of the tool. Given that these (and related) transformations need to be managed during the use of tools, it is worth asking *where* these transformations might be represented? If they are “represented” simply during the performance of an action, and arise from the moment-to-moment correction of the action, then one might not expect to see anticipatory effects. On the other hand, if there is evidence of anticipation (of the consequences of any of these transformations) then this implies a need to represent the consequence and the question remains, where does this representation reside and what form does it take?

Before considering the questions of transformations, it is worth repeating some of the observations from these studies regarding expertise. For example, Roux et al. (1995) showed that expert craftsmen (making stone or glass beads) showed significantly less inter- and intra-individual variations in performance than less experienced workers. Similarly, Biryukova and Bril (2008) showed that expert knappers used a larger repertoire of joint angle combinations than their less qualified colleagues (who tended to demonstrate more rigid behavior), and Bril et al. (2010) showed that experts showed a lower variability in kinetic energy compared to intermediates and novices. In related work, Vernooij et al. (2012) explored learning in a task involving the use of a 300-g hammer-stone. Analysis of motion tracked during the performance of this task showed inter-individual differences in the ways in which joint angles were combined to strike a particular type of blow and that these combinations changed during the course of the study. The analysis of learning to use such a hammer suggested that participants were only able to modify one parameter (relating to joint angles or impact force) but not both at the same time, until they had gained proficiency in the task.

In a series of experiments comparing expert, intermediate and novice users of stone hammers (in flint-knapping tasks conducted in the laboratory), Bril et al. (2010) identify three primary parameters that seem to contribute to the dynamics of tool use in this context. The first are Control Parameters, such as the velocity with which the hammer stone approached the target. The study showed that, in general, novices appreciated the need to control velocity but were not able to control this efficiently (this finding is supported by the work on Vernooij et al., 2012, discussed above). Thus, we would expect greater variability in the novice performance on these control parameters; as Seymour (1972) put it, the expert actions would be performed in a “smoother,” “more consistent,” “more rhythmical[ly]” manner. The second set of parameters considered by Bril et al. (2010) are regulatory parameters, such as the trajectory followed by the hammer stone and the potential energy applied. Experts tended to show shorter trajectories and smaller ratios between parameters. In Seymour’s (1972) terms, this shows how experts are able to use a “higher degree of temporal organization” and also to make “better use of the sensory data” in managing their actions. As Bril et al. (2010) note, *“In the present task, the velocity of the hammer had to be controlled to produce the required kinetic energy in relation to the mass of the hammer. This was achieved by concurrently changing the trajectory, the amplitude of the movement, and the muscular force. In this perspective, the movement became meaningful only in relation to the production of functional parameters at the level of the task, which allowed for movement flexibility as long as the task requirements were fulfilled.”* (Bril et al., 2010, p. 837). This quotation introduces the third parameter, the Functional parameter, such as kinetic energy, which experts appear to hold constant and aim to apply the lowest kinetic energy that is sufficient for the task. As the experiments involved presenting participants with hammers of different weights and requiring them to produce flakes of different sizes, one can assume that all participants would be able to discern changes in hammer weight or task demands (in terms of flake size), but the results suggest that a characteristic of expertise (which was not available to the novices) was the ability to respond to “nested relationships” (Wagman and Carello, 2003) between weight of hammer and size of flake to produce. The ability to appreciate these “nested relationships” allowed the experts to interpret the constraints placed on them by the person–environment–tool–object relationships and respond to these in ways that the novices could not. So, we return to the question of *where* these constraints might be represented? One possibility (implied by Seymour, 1972 and mooted by Bril et al., 2010) is that the initial representations involve Functional parameters which are learned and then adapted to changes in context.

In his discussion of dexterity, Bernstein (1967) highlighted that the main determinant was not bodily movement so much as the capability to respond to changes in the conditions surrounding the person. Bernstein’s (1967) notions of tool use, in terms of dexterity, relate to the quotation from Simon (1969) at the start of this paper. The manipulation of tools is rarely an end in itself but is performed with the intention of shaping objects in the environment. The actions performed lead to changes in the objects but also indicate the intentionality of the tool user (providing they have sufficiently dexterity in their use of the tools). The expert tool user thinks through the tools that are used because the actions performed with the tools shape the environment in such a way as to solve the problems that it presents and in such a way as to produce the results that the tool user desires. The action performed with the tool also creates the opportunities for the next action; and this, in turn, reflects the type of grip and posture which the tool user adopts. In this way, grip and posture (in holding and using a tool) indicate the chosen solution to the problem that the tool user is solving.

In much the same way that Rosenbaum et al. (2012) speaks of end-state comfort (and the ways in which a posture anticipates a particular end-state following the movement), so we can think of the ways in which the tool user is continually seeking to adapt their current motions in anticipation of subsequent motions and states of the object. For this paper, we take this to mean that the skilled tool-user is better able to coordinate the person–environment–tool–object system and to anticipate how changes in this system require adaptation of activity. This real-time adaptation need not imply internal representation of either task dynamics or some form of “motor program.” Rather, the expert is able to produce movements which are coordinated to task goals (being more efficient and economical in terms of energy use). In a sense, expertise is the practiced adaptation of intrinsic dynamics to task dynamics (where task dynamics are defined by the person–environment–tool–object system) so that changes in task constraints and affordances can be appropriately responded to through subtle tuning of actions. This implies that experts are able to modify the pattern of activity without necessarily impairing the functional impact of the activity. We do not believe that experts need to possess, or even represent, these various patterns of activity but rather these arise on-the-fly during the coordinated control of limbs holding and controlling tools.

## STUDIES USING SENSORS FITTED TO THE HANDLES OF TOOLS

**FIGURE 2:**
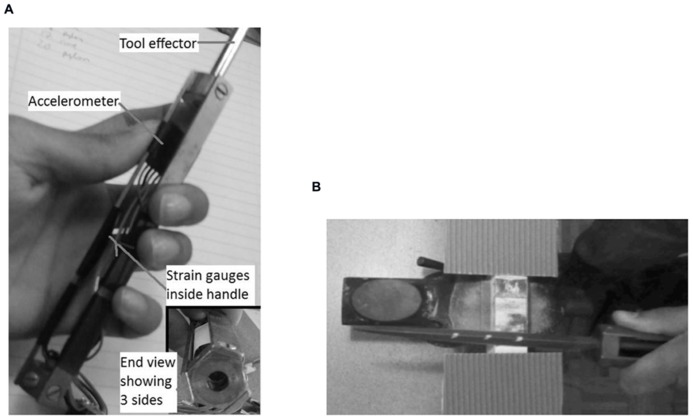
**(A)** An instrumented handle and **(B)** using a file in an instrumented handle to remove paint from a piece of wood.

In order to explore these questions of dynamics, we have been exploring ways in which to capture behavior in the field (or, at least, in laboratory and workshop settings which are as close to the field as possible). This has involved designing and developing handles which combine different types of sensor to capture the actions a person performs. Often such data are collected from using camera systems with markers on the person. While these can be very accurate, they are not easy to use in the field. Thus, it makes more sense to instrument the person or their tools in order to collect data *in *situ. We have taken the lead from Bril and her colleagues (discussed in the previous section) to instrument our tools (**Figure 2**). In our work, strain gages are used to capture force applied to the handle and a three-axis accelerometer is used to capture motion (Parekh and Baber, 2010 for a description of the design of these handles).

**FIGURE 3:**
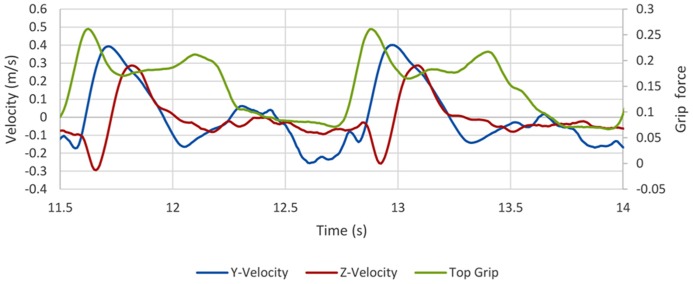
**Example of data collected from experienced silversmith using a file.** The data were sampled at 120 Hz. Velocity is derived from accelerometer data, de-trended using a moving average of 100 samples and grip force is the average output from the Analog to Digital Converter (ADC). The *Y* velocity line describes anteposterial motion, the *Z* velocity line describes vertical displacement, and the top grip describes the force applied to the top of the handle (pressing down on to the metal).

In order to appreciate how experience in using a given tool can shape activity, **Figure 3** presents an extract of recordings (from a three-axis accelerometer and strain gages integrated into the handle of a jeweler’s file) of an experienced silversmith filing the edge of a metal strip. **Figure 3** shows three filing strokes over the course of 2.5 s. Each stroke (occurring at approximately 11.6, 12, and 12.9 s) is indicated by an increase in the *y*-velocity data. There are two types of stroke here: rapid (at 11.6 and 12.9 s) in which the file in moved rapidly across the metal, and slow (12 s) in which the file is drawn more slowly over the metal. During each stroke, there is downward pressure on the file (indicated by the decrease in *z*-velocity data and increase in “top grip” force applied to the top of the handle). Immediately following the stroke, the file is lifted up (increase in *z*-velocity) and brought back to the starting point. Prior to the next stroke the file is adjusted and aligned with the metal (which takes around 1 s), which involves little change in grip force applied and z-velocity. The top grip loosens as the file position is reset for lifting the file off the object (movement in the *z* direction); the expert user only applies force on the forwards motion. This action is partly dictated by the file being used and partly by the results that the tool user intends. As the expert said, you can remove metal easily enough but you can’t put it back. So filing is about removing sufficient (but not too much) of the metal. Furthermore, the metal being worked (copper in this instance) could easily be dulled if too much of the upper surface was removed, and so filing was also a matter of retaining the luster of the metal. Such knowledge can affect the way in which the tool is wielded and influence the outcomes that one might expect when using the tools. 

In another study, we asked novice users of a file to remove paint from a piece of wood. **Figure 2B** shows the task being performed. There are three dots painted on the top of the file and participant was instructed to ensure that the file was kept between the first and second, or the first and third dots.

**FIGURE 4:**
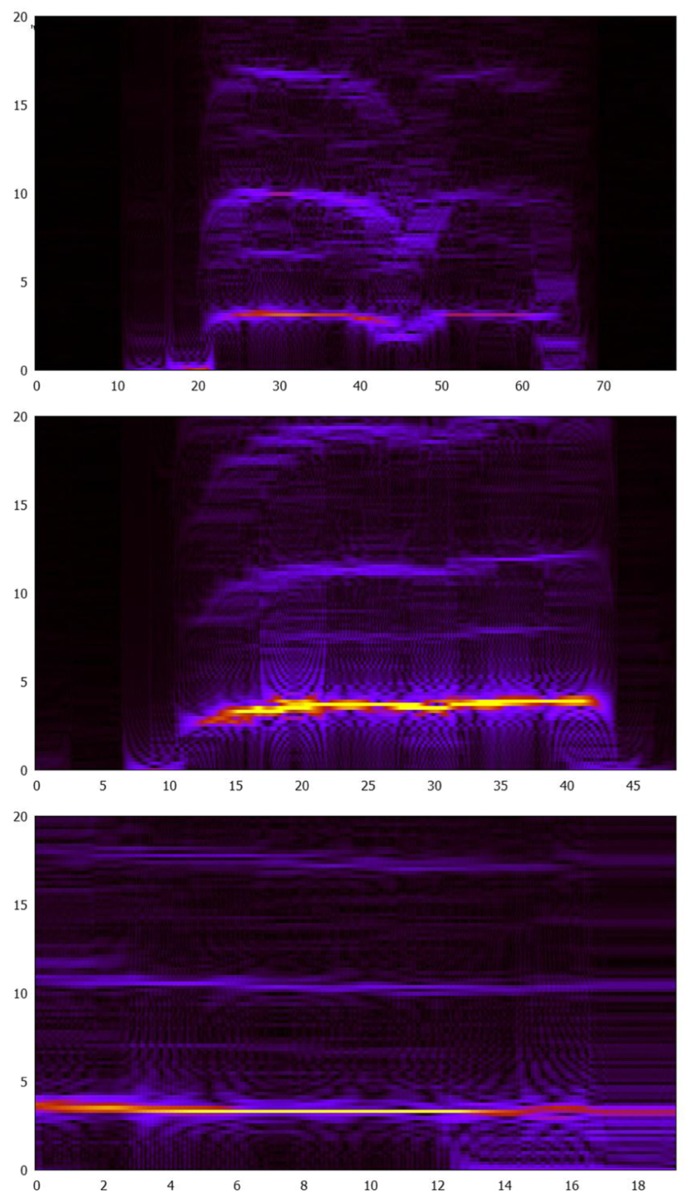
**Spectrograms from three participants performing one of the “paint removal” tasks.** The spectrogram of frequencies 0–20 Hz over time for *y*-movement (i.e., back and forth motion of the file across the wood), moving between the 1st and 3rd dots on the file with the goal of removing two layers of paint.

Contrasting three people performing the filing task (**Figure 4**), we can see that while the main activity (yellow on the spectrographs) occurs at similar frequencies, the harmonics vary. These variations might reflect differences in strategy. We would expect to see harmonics from these data due to the periodicity of the repetitive motions employed. This also suggests that differences in performance can be captured through a better appreciation of dynamics and, potentially, following the lead of Bernstein (1967), can be reflected in the conservation of energy of the tool users.

The raw accelerometer data were integrated to produce velocity, on which we applied a Fourier transform to determine the fundamental frequency of the filing motion. **Table 1** suggests that the main determinant of this fundamental frequency is not the tool-specific goal to keep the two dots inside the wood, but the task-specific goal to remove one or two layers of paint.

**Table 1:** Comparing fundamental frequency of filing for task and tool-directed goals.

Task goal	Filing white paint down to show red paint		Filing white paint down to show bare wood	
Tool-directed goal	Keep file between dot a and dot b	Keep file between dot a and dot c	Keep file between dot a and dot b	Keep file between dot a and dot c [7pt]
F0	6.201 Hz	5.518 Hz	3.174 Hz	3.467 Hz

### CULTURAL AFFORDANCES

In this section, we turn our attention to the broader question of cultural effects in tool use. For the sake of the discussion, we restrict ourselves to the simple assumption that cultural constraints can have a bearing of the experiences that people might have with specific types of tools and, in particular, can serve to define acceptable or proper ways in which particular artifacts are used. Thus, one question that can be used to address the issue of “culture” in tool use is to ask how should one *properly* use cutlery, such as a spoon, knife or fork?

In their study of eating (kale or water) with a spoon, van der Kamp and Steenbergen (1999) used video-based motion tracking to record arm motion. The likelihood of spilling the contents of the spoon (kale or water) when it was moved from bowl to mouth increased the number of corrective sub-movements made during the action which affected the kinematic profile of the movement. The contents of the spoon also affected head motion. Participants were more likely to move their head towards the spoon when it contained water which, in turn, shows how the coordination of the motion system (i.e., contents–spoon–hand–arm–head) changes in response to task demands. Interestingly, the study also hinted at variation in “eating styles” which reflected individual differences in performance. We are interested in how these “eating styles” might also reflect cultural responses to cutlery and how culture defines the “proper” way to use an item of cutlery. Of course, the use of the word “properly” is deliberately provocative and culturally loaded. At one level, “proper” use could simply mean that food is moved from plate to mouth in a controlled manner, in sufficient quantities to make it easy to eat. At another level, “proper” use could relate to various social mores and rules of etiquette in terms of how the knife and fork are held and moved, and how much food is held on the fork or put into the mouth. For example, in her discussion of using forks, Visser (1991) contrasts the “English” style (of eating from the back of the fork tines and holding the knife in the other hand) with the “American” style (of eating from the bowl of the fork and swapping, or “zig-zagging” fork and knife).

**FIGURE 5:**
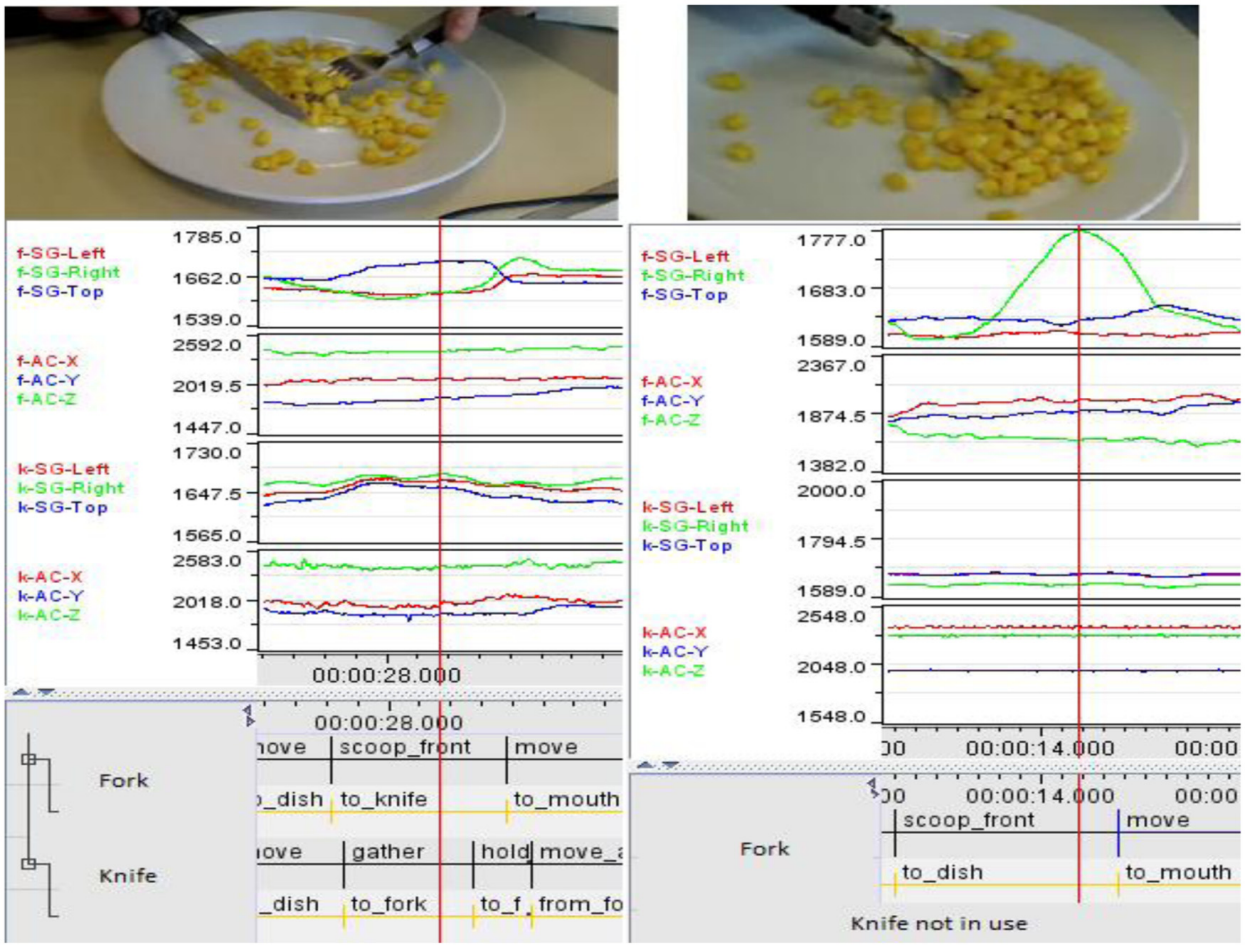
**Comparing English (left) and American (right) cutlery use**.

**FIGURE 6:**
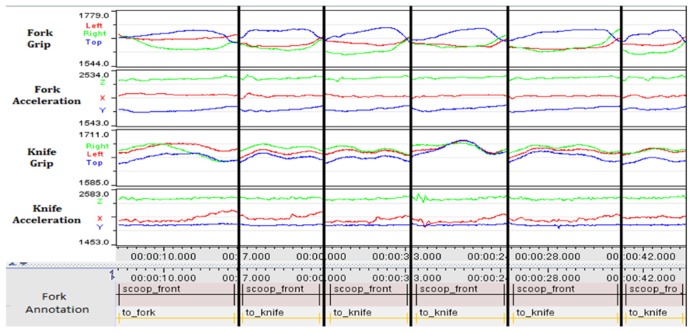
**Consistent “English” use (over six separate attempts as indicated by thick lines between each attempt).** The pattern of grip force applied (particularly to the fork handle) and the smoothness of the fork’s accelerometer trace show how the experienced participant’s repetitions are consistent and reflect a well-practiced motion.

**FIGURE 7:**
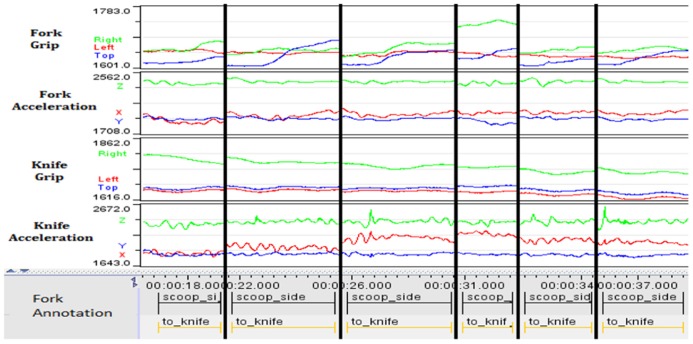
**Variable “English” use (over six separate attempts).** The *inexperienced* participant shows large variation in grip force and accelerometer trace for the fork. During the task, his preferred approach was to tilt the fork on its side and move it towards the kernels, using the knife as a stop. He then held the fork at an angle and used the knife to keep the kernals pressed to the tines as he lifted both knife and fork. There is less correlation shown between grip and activity from the knife, which is being pushed on to the top of the fork, and, particularly towards the right of the graphs, the fork is held with force primarily only on two sides of the handle as opposed to a full grip.

We asked participants, using a knife and a fork (fitted to our instrumented handles), to perform a somewhat unusual version of “eating.” The task goal required participants to lift a forkful of sweet-corn to their chin. This breaks down into: “load fork’, “lift fork’, and “terminate” (e.g., most participants simply tipped the fork to drop the sweet-corn back onto the plate). The “English” or “American” styles outlined above are illustrated by **Figure 5**. In order to consider variation, we selected one participant who was familiar with the “English” style (**Figure 6**) and one of the participants who had never used cutlery in this manner (**Figure 7**).

**Figures 6** and **7** show that variability in the data from the inexperienced user are consistently higher than the experienced user for both grip and accelerometer data. This echoes the earlier findings relating the variability in “skilled” performance. Rather than the “skill’, in this case, being the result of instruction, training and practice (as one might expect in the use of hand-tools), these results hint that enculturation and exposure to particular beliefs about appropriate use of cutlery can have an impact on the ease with which these artifacts are manipulated in different ways.

## DISCUSSION

We use tools to solve the problems that objects in the environment present to us. This is an obvious statement but hides a couple of points which are worth noting. The first is that intention which underlies the use of the tool combines a task goal with the affordances of the tool–object interface, and the constraints of the person–environment–tool–object system. This means that “cognition” becomes the active response to the affordances of the interaction between tool and object in terms of the task goal that the user is seeking to achieve. Taking Gibson’s notion of complimentarity, we can say that the dynamic aspect of this activity continually shapes the actions of the person as much as it shapes the state of the object. In other words, the states of the object, environment, tool, and person become combined to form the focus of action and, by implication, to help frame and reframe the task goal. One might expect the task goal to be kept constant during the performance of the task. However, our discussions with, and observations of, expert jewelers suggests that this not entirely the case. While the high-level objective might remain the same (e.g., produce a ring of a particular size set with a particular stone), the development of the “plan” to achieve this goal adapts to the state of the metal and the performance of the task. Thus, the task goal would appear to follow the notion of “situated action” (Suchman, 1987) which changes with context. This raises the second point, that, the focus of action is context-dependent and the context is continually changing. So, tool use is enactive, embedded and embodied.

The comparisons of experienced and inexperienced users of tools (and cutlery) considered in this paper show that expertise not only involves less variability in physical performance but also better control of energy expended in the performance of a given task with a given tool. We believe that this points to the well-known assertion that the expert develops a “feel” for the tool, and often prefer to use their own tools for particular tasks because these have become very familiar to them. Indeed, a potential problem that we face with the instrumented handles that we use is that these feel different from those that the experienced tool users prefer. Anecdotally, only the experienced tool users commented on the feel (weight, balance, material) of these handles during the data collection.

The skilled craft-worker will often speak of the tool becoming part of the body, and the feeling of manipulating the tool being akin to simply moving the hand in which the tool held. For some writers, this implies that the tool can be considered as a physical extension of the person and that, therefore, motor control becomes a matter of adapting to the added potential of the “extended-limb.” However, rather than simply being a matter of planning movement with the addition of the tool, it is plausible to suggest that the tool changes the perception of space around the tool user (Maravita et al., 2002). *“People who use tools…build an increasingly rich implicit understanding of the world in which they use the tools…” *[Cutler, 1994, p. 80]. In her discussion of representations in tool-use, Massen (2013) emphasizes the need to appreciate how tools become part of the peripersonal space of the user, such *that “there is no need to distinguish between external goal locations to which the tool has to be moved and the locations to which the bodily effector has to be moved.”* (p. 2). While this makes sense when considering movements with tools, it overlooks an equally important aspect of the skilled craft-worker. The reason that the tool feels as if it is part of the person is because it “disappears” from attention which becomes more and more focused on the object being worked on. This suggests that, rather than the tool being an extension of the body, it makes more sense that the tool creates a focus of attention – with the sense that the tool’s movement becomes so central to attention that the control of the limb operating it becomes less important. This suggests that, rather than considering the tool-hand combination, it is more important to consider the tool-object combination because this is where the skilled practitioner is attending.

The use of tools, by experts, seems to involve anticipatory, feed-forward control of movement (as well as rapid and efficient use of feed-back through all of their senses) in which subtle adjustments in the manipulation of the tool are performed in order to effect desired changes in the object being worked on. Not only does this explain the minimal variability but also highlights the central question of this paper; if so much of the activity of the expert tool user is anticipatory, how are these anticipations represented? We propose that it is not sufficient to only look in the brain of the expert tool user to discover these representations. Even if there are regions which are active under specific conditions, the skill of the expert tool user comes from the ability to control their activity with sufficient spare capacity to cope with future demands and to respond to the changing context in which they are using the tools to effect changes in the object being worked on. The idea that the environment (and the objects it contains) can be interpreted in different ways, suggests that these become “external representations” to which the person responds. Response is partly a matter of knowledge, skill and ability of the person, partly a matter of fit between action and environment and partly a matter of the nature of the environment and the objects it contains. As the person focuses on specific aspects, which are relevant to the task (of shaping a piece of metal or arranging tools in a workspace) so these aspects become the cognitive space in which subsequent decisions are made. Tool use, as a form of problem solving, becomes a matter of making these decisions as the cognitive space changes; and a means of acting upon the cognitive space to create new opportunities. This further suggests that much of the activity which is assumed to be “feed-forward” (in the sense that there needs to be a model which guides behavior) could be explained by fast-acting, negative feedback loops (integrated across several sensory modalities) which support moment-by-moment correction through solving the inverse kinematics problems of positioning a given tool in a given position in order to effect change in the object being worked on.

We believe that much of the “representation” drawn upon in the use of tools can be in the form of external representations (the objects and tools in a given environment, particularly in support of the situated action of ongoing planning in tool use) and in the form of coordinative structures (the control and management of physical activity, particularly in terms of feed-forward control of movement and use of feed-back from the results of the movement). In other words, following the lead of Riccio (1993) and the more detailed arguments of Chemero (2009) an internal representation is not necessary for the control, coordination and (we propose) planning of tool use because it is sufficient for the tool user to have the ability to perceive the state of the object on which she works and to manipulate the tool in order to produce a particular pattern of perceptions (and, in this case, we suggest that these patterns are equally as likely to be olfactory, haptic, and auditory as visual). This ability becomes manifest only during the performance of a person–environment–tool–object system (echoing Butler’s claim that “strictly speaking, nothing is a tool except during use”) and this system can be described using System Dynamics, in which the systems goal is the optimization of specific movement parameters in order to produce an effect on a given object. This reduces the need for there to be internal representations *per se *(see also Barrett, 2011). Furthermore, any “representation” that the tool user employs is likely to spread across the entire nervous system rather than solely in regions of the brain. From this, the strong and compelling evidence accumulated from the activation of specific regions in the brain is taken to indicate the result rather than the cause of tool using behavior (whether observed, imagined, or performed) which arises from the recruitment and activation of coordinative structures (Bernstein, 1967) through task-specific devices (Beek and Bingham, 1991). While our paper has not sought to present evidence in support of this claim, we believe that this statement helps to bring together the ongoing work that we have reviewed and raises the opportunity to develop testable hypotheses for future exploration of the ways in which people use tools.

## Conflict of Interest Statement

The authors declare that the research was conducted in the absence of any commercial or financial relationships that could be construed as a potential conflict of interest.
